# 

**Published:** 1905-01-07

**Authors:** 


### NOTICE TO CORRESPONDENTS.

All MSS., Letters, Books for Review, and other matters intended for the Editor should be addressed The Editor, "The Hospital," The Hospital Buildings, 28 & 29 Southampton Street, Strand, W.C.

The Editor cannot undertake to return rejected MS., even when accompanied by stamped directed envelope.

**Notice to Advertisers and Subscribers.**

All Advertisements, Orders for Copies of The Hospital, and Business Communications should be addressed to **THE MANAGER (Not to the Editor),** The Hospital, 28 & 29 Southampton Street, Strand, London, W.C.

The Cost of Subscription, post free, is as follows:—Per week, 3½d.; per quarter, 3s. 3d.; per half-year, 6s. 6d.; per annum, 13s. Foreign and Colonial:—Per annum, 19s. 6d., payable, in all cases, in advance.

**NOTICE.—Vol. XXXVI. of "The Hospital" (April to September 1904), in handsome cloth binding, is now on sale at the office of this Journal, price 10s. 6d. Cases for binding the Numbers contained in this Volume 2s. Index to Vol. XXXVI., 3½d., post free.**

**The Monthly part for January is now ready. Price 1s., by post, 1s. 3d.**

### MEDICAL APPOINTMENTS.

W. I. T. Baker, L.R.C.P. and S. Edin., L.F.P.S.Glasg., Medical Officer of Health, Winterton Urban District. G. E. Gask, F.R.C.S., Assistant Surgeon to the Metropolitan Hospital. E. M. Goldie, M.D.Edin., Public Vaccinator to the Woodford District of the West Ham Union, vice Dr. Sayres, resigned. J. Hill, L.R.C.P. and S. Edin., Certifying Factory Surgeon for the Miltown Malbay District, co. Clare. S. S. Hoyland, M.D., District Medical Officer of the Samford Union. R. G. Johnson, M.B., Certifying Factory Surgeon for the Poole District, Dorsetshire. C. P. Lapage, M.B., Ch.B.Vict., Senior Resident Medical Officer to the St. Mary's Hospital, Manchester. S. C. Legge, M.R.C.S., L.R.C.P., Medical Officer of the Workhouse and Cottage Homes, Worcester. T. H. Livingstone, F.R.C.S., M.D.Edin., Honorary Assistant Surgeon to the Hospital for Children, Newcastle-upon-Tyne. T. G. Mathews, M.D.Edin., Certifying Factory Surgeon for the Kirby Lonsdale District, Westmorland. Arthur Matthey, M.R.C.S., L.R.C.P., Medical Officer in Charge of the Port Morant District of British Guiana. Florence M. S. Price, M.B., Ch.B.Edin., House Physician to the Swansea General and Eye Hospital. H. Samuel, M.R.C.S.Eng., Resident Medical Officer to the Cardiff Infirmary. Gustave Schorstein, D.M., M.A.Oxon., F.R.C.P., Physician to the Brompton Hospital.

**"The Hospital" Institutional Library.**

"Hospitals and Asylums of the World." 4 Vols., with a Portfolio of Plans, complete £12 12s.
"Burdett's Hospitals and Charities, 1904." 5s. net.
"Burdett's Official Nursing Directory." 3s. net.
"Cottage Hospitals, General, Fever, and Convalescent." 10s. 6d.
"Uniform System of Accounts for Hospitals and Public Institutions." New Edition. 4s. net.
"Hospital Expenditure: The Commissariat." 2s. 6d.
"A Legal Handbook for the Use of Hospital Authorities." 1s. 6d.
"The Cottage Hospital Case Book or Register of Patients." 10s. and 12s. 6d.

All these are published by the SCIENTIFIC PRESS, Ltd., and may be obtained through any bookseller, or direct from the publishers, 28 and 29 Southampton Street, Strand, London, W.C.

### A Dozen Special Text-Books for Nurses.

| | | |
|---|---|---|
| 1. | A COMPLETE HANDBOOK OF MIDWIFERY. By Dr. J. K. WATSON. Specially designed to meet the requirements of the Central Midwives Board. Illustrated with 143 Diagrams, &c. | 6/- net, 6/4 post free. |
| 2. | HOW TO BECOME A CERTIFIED MIDWIFE. By E. L. C, APPEL, M.B. | 1/- net, 1/1 post free. |
| 3. | HOSPITAL SISTERS AND THEIR DUTIES. By EVA C. E. LÜCKES. Third Edition. | 2/6 post free. |
| 4. | OPHTHALMIC NURSING. By SYDNEY STEPHENSON, M.B. New and Revised Edition. Illustrated. | 3/6 net, 3/10 post free. |
| 5. | NURSING IN DISEASES OF THE THROAT, NOSE, & EAR. By P. MACLEOD YEARSLEY. Illustrated. | 2/6 post free. |
| 6. | ART OF MASSAGE. By Mrs. CREIGHTON HALE. Second Edition. Illustrated. | 6/- post free. |
| 7. | MASSAGE AND MEDICAL GYMNASTICS, with Swedish and Schott (Nauheim) Movements. By AXEL V. GRAFSTROM, M.D. Illustrated. | 2/6 post free. |
| 8. | ART OF FEEDING THE INVALID. New and Popular Edition. Bound in cloth. | 1/6 post free. |
| 9. | PRACTICAL GUIDE TO SURGICAL BANDAGING AND DRESSINGS. By WM. JOHNSON SMITH, F.R.C.S. Suitable size for the apron pocket. Profusely Illustrated. | 2/- post free. |
| 10. | THE EXAMINATION OF THE URINE. By J. K. WATSON, M.D. | 1/- net, 1/1 post free. |
| 11. | THE MODERN NURSING OF CONSUMPTION. By Dr. JANE H. WALKER. | 1/- net, 1/2 post free. |
| 12. | ON PREPARATION FOR OPERATION IN PRIVATE HOUSES. By a SURGEON. | 6d. post free |

**THE SCIENTIFIC PRESS, LTD.** *Publishers of Nursing Text-Books, Charts, &c., of all descriptions.* 28 & 29 Southampton St., Strand, London, W.C.

